# Normative measurements of the superior oblique and inferior oblique muscles by magnetic resonance imaging

**DOI:** 10.1007/s00276-022-02915-w

**Published:** 2022-03-08

**Authors:** Khizar Rana, Valerie Juniat, Aaron Rayan, Sandy Patel, Dinesh Selva

**Affiliations:** 1 Department of Ophthalmology and Visual Sciences, University of Adelaide, North Terrace, Adelaide, SA 5000 Australia; 2 South Australian Institute of Ophthalmology, Royal Adelaide Hospital, Port Road, Adelaide, SA 5000 Australia; 3 Department of Medical Imaging, Royal Adelaide Hospital, Port Road, Adelaide, SA 5000 Australia

**Keywords:** Superior oblique, Inferior oblique, Magnetic resonance imaging

## Abstract

**Purpose:**

Normative oblique muscle data may help to diagnose pathological enlargement of the oblique muscles. We aim to describe the normative values of the superior and inferior oblique muscles in an Australian cohort on T1-weighted MRI and fat suppressed contrast enhanced T1-weighted MRI.

**Methods:**

A retrospective review of patients who underwent 3 T orbital MRI. The healthy orbits were used to conduct measurements in patients with a unilateral orbital lesion. The maximum diameters of the superior and inferior oblique muscles were measured on coronal planes. The diameter was measured perpendicular to the long axis of the muscles.

**Results:**

The normal measurements (mean ± SD) on fat suppressed contrast enhanced T1-weighted MRI: superior oblique, 3.0 ± 0.5 mm and inferior oblique, 2.7 ± 0.5 mm. On T1-weighted MRI: superior oblique, 2.8 ± 0.5 mm and inferior oblique, 2.5 ± 0.4 mm. In patients who had both sequences performed, the superior and inferior oblique diameters were significantly higher on the fat suppressed contrast-enhanced T1-weighted MRI than the T1-weighted MRI sequence (*p* < 0.01).

**Conclusion:**

Oblique muscle enlargement may be seen in a range of orbital diseases. These data may help in diagnosing oblique muscle enlargement. In addition, variations in the measured muscle diameters can be seen according to the scan sequence that is used.

## Introduction

The oblique muscles may be enlarged in a range of inflammatory, neoplastic, and infective conditions. These include orbital lymphoma; inflammatory conditions, such as thyroid eye disease (TED), myositis, Sarcoidosis, and IgG4-related ophthalmic disease; and infective conditions including pyomyositis and cysticercosis [1, 4, 6, 7, 9, 11, 12, 17, 20].

Studies reporting normative data on the oblique muscles are limited. Previous studies have conducted oblique measurements on standard T1-weighted MRI [16, 18]. Fat-suppressed contrast enhanced imaging is the preferred imaging modality for evaluating inflammatory and neoplastic conditions affecting the extraocular muscles. It is likely that the measurements vary according to scan sequence that is used.

We describe and compare the normative values of the superior and inferior oblique muscles in an Australian cohort on T1-weighted MRI and fat suppressed contrast-enhanced T1-weighted MRI.

## Methods

The study was approved by the Central Adelaide Local Health Network ethics committee and adhered to the principles of the Declaration of Helsinki.

This was a retrospective review of patients who underwent high-field (3 Tesla; 3 T) magnetic resonance imaging of the orbits. Patients with conditions known to affect bilateral orbits (e.g., TED, trauma), high myopia, prior orbital surgery or poor scan quality were excluded. The normal, healthy orbits were used to conduct measurements in patients with unilateral orbital disease.

**Fig. 1:**
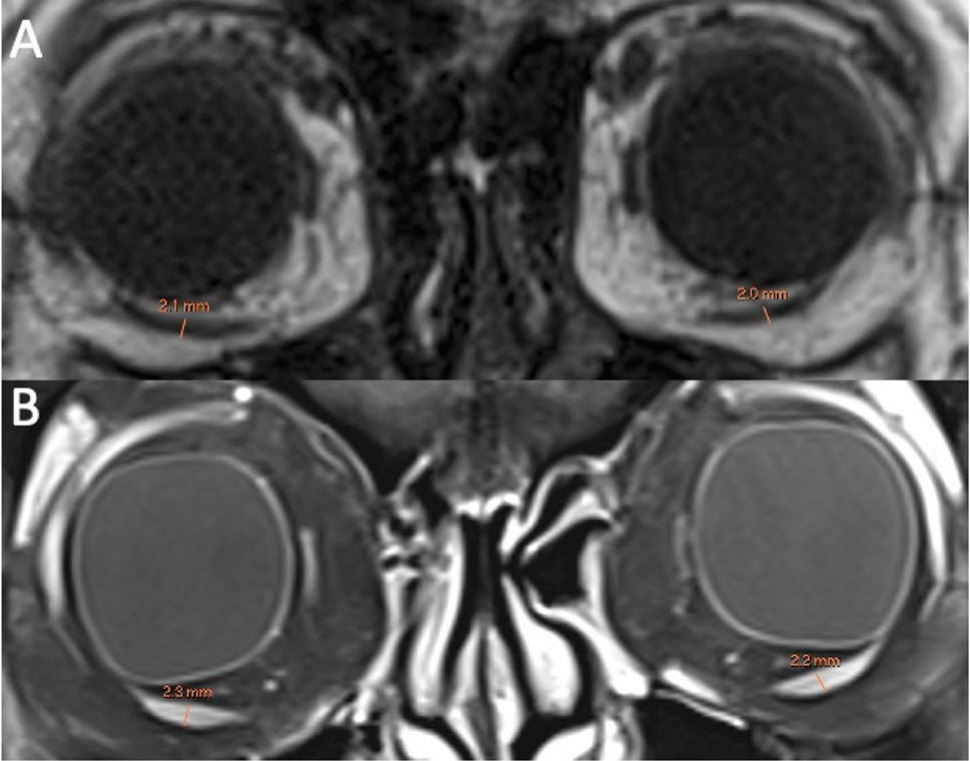
Coronal T1-weighted MRI (**A**) and fat suppressed contrast-enhanced T1-weighted MRI (**B**) showing the measurements of the inferior oblique muscle

**Fig. 2:**
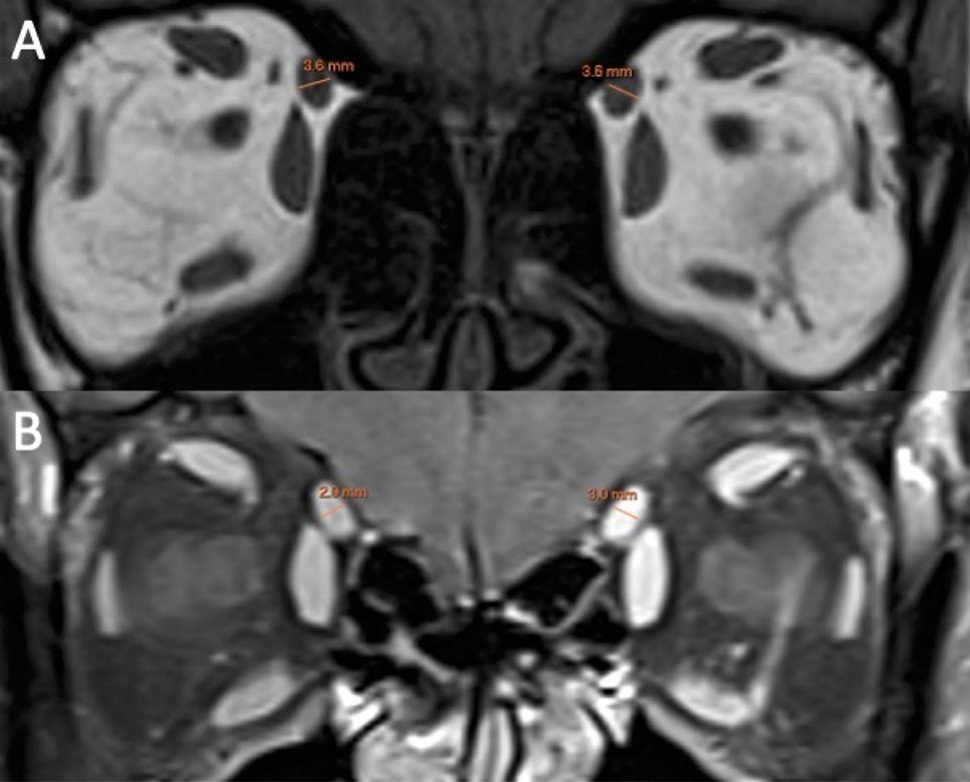
Coronal T1-weighted MRI (**A**) and fat suppressed contrast-enhanced T1-weighted MRI (**B**) showing the measurements of the superior oblique muscle

Patients were evaluated using Magnetom 3 T Skyra scanner (Siemens AG, Munich, Germany) with a conventional turbo spin-echo sequence (TR/TE, 500/15; field of view, 200 × 200 mm; matrix, 512 × 512; slice thickness 3 mm). Contrast enhanced images were obtained after intravenous administration of a standard weight-based dose of gadolinium. The maximum diameters of the superior and inferior oblique muscles were taken on coronal planes (Figs. 1, 2). The diameter was taken perpendicular to the long axis of the muscles. All measurements were performed on high resolution picture archiving and communication system (PACS).

Statistical analysis was performed using Stata 13.0 (StataCorp, College Station, Texas). In patients who had both normal orbits, only the right orbit was used to avoid violating the assumption of independence. Shapiro–Wilk tests for the both the SO (*W* = 1.00, *p* = 0.988) and IO (*W* = 0.99, *p* = 0.208) did not show evidence of non-normality. The mean values of the muscle thickness were calculated and presented as mean ± standard deviation. The independent samples *t* test was used to compare the data from male and female patients. Pearson’s correlation coefficient was used to assess the correlation between age and muscle thickness. The paired *t* test was used to compare the diameters from the two different MRI sequences. Bland–Altman analysis was used to assess for interobserver and intraobserver reliability [3]. Thirty scans were assessed by a second reviewer (AR) and fifteen scan measurements were repeated by the first reviewer (KR). The reviewers were blinded to the original results. The criteria for significance was set at *p* < 0.05.

## Results

### Fat suppressed contrast-enhanced T1-weighted MRI

141 orbits from 141 patients (64 male, 77 female) had fat suppressed contrast enhanced T1-weighted MRI. The mean age of these participants was 58 ± 18 years (20–94 years). The mean diameters of the superior and inferior oblique muscles for all participants are given in Table 1. There was no significant difference in the size of muscles between sexes. No significant correlation was seen between age and superior oblique (*r* =  − 0.13, *p* = 0.12) or inferior oblique thickness (*r* =  − 0.09, *p* = 0.28).

**Table 1:** Normative orbital measurements on fat suppressed contrast-enhanced T1-weighted magnetic resonance imaging in millimetres

Muscle	Total	Male	Female	*p* value
Superior oblique	3.00 ± 0.52	3.04 ± 0.52	2.97 ± 0.51	0.43
Inferior oblique	2.69 ± 0.48	2.76 ± 0.53	2.63 ± 0.44	0.11

### T1-weighted MRI

There were 84 orbits from 84 patients (40 male, 44 female) with a mean age of 56 ± 18 years (20–87 years) who had T1-weighted MRI scans. The mean diameters of the superior and inferior oblique muscles for all participants are given in Table 2. No significant difference was observed for the superior or inferior oblique thickness between males and females. No significant correlation was seen between age and superior oblique (*r* =  − 0.11, *p* = 0.34) or inferior oblique thickness (*r* =  − 0.09, *p* = 0.40).

**Table 2:** Normative orbital measurements on T1-weighted magnetic resonance imaging in millimetres

Muscle	Total	Male	Female	*p* value
Superior oblique	2.80 ± 0.52	2.83 ± 0.54	2.78 ± 0.51	0.69
Inferior oblique	2.47 ± 0.42	2.50 ± 0.42	2.43 ± 0.42	0.46

### Fat suppressed contrast-enhanced T1-weighted MRI vs T1-weighted MRI

There were 71 orbits from 71 patients (34 male, 37 female) who had both scan sequences performed. The mean age of these participants was 58 ± 17 years (20–87 years). The muscle diameters for the two sequences are presented in Table 3. The superior and inferior oblique diameters were significantly higher on the fat suppressed contrast-enhanced T1-weighted MRI than the T1 weighted MRI (*p* < 0.01).

**Table 3:** Normative orbital measurements on fat suppressed contrast-enhanced T1-weighted MRI compared to T1-weighted magnetic resonance imaging in millimetres

Measurement	T1w-MRI	T1 FS CE	*p* value
Superior oblique	2.82 ± 0.52	3.02 ± 0.55	< 0.01
Inferior oblique	2.47 ± 0.39	2.64 ± 0.41	< 0.01

*T1 FS CE* T1-weighted fat suppressed contrast enhanced MRI

The normal ranges of the oblique muscles on T1-weighted MRI across different studies are given in Table 4. Bland–Altman analysis showed agreement for the intraobserver and interobserver reliabilities with 90–100% of points lying within the limits of agreement.

**Table 4:** Normative ranges of the oblique muscles on T1-weighted magnetic resonance imaging

Study	Superior oblique		Inferior oblique	
	Mean (mm)	Range (± 2 SD)	Mean (mm)	Range (± 2 SD)
Our study	2.8	1.8–3.8	2.5	1.6–3.3
Ozgen et al. [12]	3.2	2.4–4.1	NR	NR
Shen et al. [10]	2.2	0.8–3.6	NR	NR
Sabundayo et al. [11]	3.0	NR	NR	1.9–4.1

*NR* not reported

## Discussion

We present the normative data on the oblique muscles in an Australian cohort on two different MRI sequences. Amongst patients who had both scans, we found that the diameters from the fat suppressed contrast-enhanced T1-weighted MRI were significantly higher than the T1-weighted MRI diameters. This is likely due to partial volume averaging at the muscle–fat interface. On a fat-suppressed contrast enhanced study, the muscle signal dominates at the muscle–fat interface, whereas the opposite can be observed in a standard T1-weighted MRI.

The upper range of the normal muscle diameters, two standard deviations above the mean, may be a suitable cutoff point to help diagnose muscle enlargement on MRI (Table 4). A diameter of greater than 4.0 mm can be considered enlarged for the superior and inferior oblique muscles. Superior oblique atrophy may be diagnosed with a diameter of less than 2.0 mm and inferior oblique atrophy with a size of less than 1.5 mm. The diameter ranges reported by Shen et al. [18] are lower than our values and those reported by Ozgen, Aydingöz [15]. This is likely because they did not measure the maximum muscle diameters and instead measured the diameters at two preselected planes 0 mm and 7 mm behind the globe.

Primary neoplasms such as orbital lymphoma or metastases from distant sites may cause oblique muscle enlargement [8, 20]. Oblique muscle enlargement has also been recognised in TED [4]. Oblique muscle enlargement may be seen without involvement of the inferior and medial recti muscles typically involved in TED [2]. Further research is required into whether oblique muscle enlargement may be a prognostic marker for disease severity in TED.

Extraocular muscle sizes may change with increasing age. Previous reports have shown a statistically significant positive correlation between age and the diameters of the inferior and lateral rectus muscles [14, 15]. However, others have failed to show any such changes [13, 16, 18]. In our study, the oblique muscle diameters were inversely correlated with age on both scan sequences; however, this did not reach statistical significance. Similarly, sex differences have been reported with males generally having larger extraocular muscle diameters than females [10, 14, 15]. Our study did not show any significant sex differences in the oblique muscle diameters.

We used a readily available and easy to perform technique to measure the diameter of the oblique muscles. The calibre tool that was used is available on the hospital’s picture archiving and communication system. The oblique muscles’ cross-sectional areas and volumes can also be measured and have been previously reported; however, these measurements require more time and expertise to perform [5, 19].

The oblique muscles may atrophy and decrease in size in certain neuro-ophthalmological conditions. The cross-sectional areas and volumes of the superior or inferior oblique muscles are smaller in cases of clinically diagnosed superior or inferior oblique palsies [5, 19]. These normative data may also help to diagnose oblique muscle atrophy using simple muscle diameters.

This study has some limitations inherent to the study design. Patients were asked to maintain forward gaze; however, the direction of gaze could not be controlled for in this study. In addition, this was a retrospective study in an Australian cohort and our data may not be applicable to other cohorts.

In conclusion, we have presented the normative data on the thickness of the superior and inferior oblique muscles in an Australian cohort. We have found significantly larger oblique muscle diameters to be reported from fat suppressed contrast-enhanced T1-weighted MRI as compared to standard T1-weighted MRI. The technique used is easily accessible and may be easily translated to a clinical setting to diagnose oblique muscle enlargement or atrophy.
